# Vesicoureteral Reflux and Duplex Systems

**DOI:** 10.1155/2008/651891

**Published:** 2008-07-17

**Authors:** John C. Thomas

**Affiliations:** Department of Urology, Division of Pediatric Urology, Monroe Carell Jr. Children's Hospital at Vanderbilt, 2200 Children's Way, Nashville, TN 37232, USA

## Abstract

Vesicoureteral reflux (VUR) is the most common anomaly associated with duplex systems. In addition to an uncomplicated duplex system, reflux can also be secondary in the presence of an ectopic ureterocele with duplex systems. Controversy exists in regard to the initial and most definitive management of these anomalies when they coexist. This paper will highlight what is currently known about duplex systems and VUR, and will attempt to provide evidence supporting the various surgical approaches to an ectopic ureterocele and duplex system and the implications of concomitant VUR.

## 1. INTRODUCTION

Less than 1% of the general population has a duplex kidney [1]. Females are affected more commonly than males and this anomaly is bilateral in 17–33% of cases [2]. VUR is the most common associated anomaly found in duplex kidneys and is present in 70% of these patients who present with a urinary tract infection [3, 4]. VUR almost always occurs into the lower-pole moiety due to its lateral displacement within the bladder. If VUR is seen in the upper-pole moiety, one must suspect a laterally displaced incomplete duplication or an ectopic orifice located within the bladder neck or urethra. This paper will review the natural history of VUR associated with uncomplicated duplex systems as well as the controversies that arise in managing reflux found in conjunction with ectopic ureteroceles.

## 2. DISCUSSION

### 2.1. VUR and duplex systems

There are certain factors that contribute to reflux resolution in single-system (SS) ureters, including patient age, grade of reflux, postnatal presentation, and the presence or absence of associated voiding dysfunction [4]. The natural history of VUR in association with duplex systems (DSs) is not completely clear. Despite several studies addressing this issue, all were limited in some way by their noncontrolled retrospective nature, patient selection or surgeon bias, and limited long-term follow-up [4]. Lee et al. followed 1/3 of their patients with VUR and DS nonoperatively, and concluded that resolution rates of low-grade (I-II/V) reflux were comparable to those seen in SS [5]. Patients with high-grade reflux were excluded from this study. A similar conclusion was noted in another study in which all grades of reflux were included. Spontaneous resolution occurred in over half of patients with grades I–III/V VUR and support consideration for initial conservative management with prophylactic antibiotics [6]. Over a two-year period of observation, Husmann et al. found that reflux resolved in 10% of patients with DS and grade II/V VUR as compared to 35% of a matched group of patients with SS; however, there were no differences in the incidence of breakthrough infections, additional renal scarring, or worsening reflux [7]. It seems clear that most patients with DS and low grades (<III/V) can be initially managed conservatively; however, VUR will likely take longer to resolve as compared to SS VUR. Clinical information concerning high-grade VUR (IV-V) and DS is lacking, although one study documented no resolution at mean follow-up of 42 months as well as an increased incidence of infectious complications, especially in young females [4].

Data from the available literature suggests that the majority of patients with DS and low-grade VUR can be initially managed with antibiotics and careful observation. Parents should be counseled that it may take longer for the reflux to resolve and young females with high-grade VUR may be at increased risk for infections. Despite these findings, the absolute indication for surgery in individuals with low-grade VUR is not different from those with SS and similar VUR, and surgical correction is successful in the majority of cases [4]. In fact, one series reported a 98% success rate for common sheath reimplantation of uncomplicated duplex systems, and concluded that the presence of a duplication anomaly does not adversely affect surgical outcome. Adequate tunnel width and long intravesical tunnels were noted to be the most important technical aspects [8]. It is important to remember, however, that complicated duplex systems associated with the need for ureteroureterostomy, ureteral tapering or tailoring, or ureteropyelostomy may carry higher complication rates than uncomplicated common sheath reimplantation.

### 2.2. Ectopic ureteroceles and vesicoureteral reflux

Duplex systems are an uncommon diagnosis causing prenatal hydronephrosis; however, when confirmed, ureteroceles are one of the most common associated findings [9, 10]. Ectopic ureteroceles can cause upper-pole hydronephrosis and obstruction, which leads to ipsilateral lower-pole reflux in 50% of cases [11]. Contralateral reflux is seen in 25% of cases and reflux into the ureterocele occurs 10% of the time [12].

The initial and subsequent management of ureteroceles has been controversial and depends on several factors, including presenting symptoms, ectopic versus orthotopic position, presence or absence of reflux, and function of the associated upper-pole moiety [11]. As the focus of this article is reflux and duplex systems, the discussion below will be limited to the management of ectopic ureteroceles in patients who present with concomitant reflux and a nonfunctioning or functioning upper-pole moiety. Management options include endoscopic puncture and decompression, a simplified upper-tract approach, namely, heminephrectomy, or complete repair including upper-pole surgery, ureterocele excision, and lower-tract reconstruction in a single setting.

In the above proposed setting, the clear indication for endoscopic decompression of an ectopic ureterocele is in a child who presents with sepsis or bladder outlet obstruction. However, in the setting of sepsis, one must open the ureterocele completely, as puncturing may not result in adequate drainage. This procedure almost invariably results in prompt improvement in patient symptoms, but the parents should be counseled that their child will require definitive reconstruction at a later date, as reflux into the upper-pole moiety is the rule, not the exception. In contrast, endoscopic puncture of an ectopic ureterocele in the nonemergent setting may also commit the patient to future reconstruction. In one series describing endoscopic puncture for ectopic ureteroceles, Jayanthi et al. reported postoperative reflux into the upper-pole moiety in 50% of cases [13]. Overall, 70% of their patients underwent open surgery with the vast majority at the level of the bladder [13]. Some have argued that initial endoscopic decompression may facilitate subsequent lower-tract surgery by reducing the size of the upper-pole ureter [14].

Upper-pole heminephrectomy can result in excellent decompression of the ureterocele and should be the procedure of choice if there is no ipsilateral lower pole or contralateral reflux [15]. Removing a functional upper pole has been advocated by some as this moiety only provides approximately 15% of total renal function at best [16]. Alternatively, one can salvage the upper pole with a ureteroureterostomy or ureteropyelostomy and subtotal ureterectomy. Success of the upper-tract approach alone without the need for subsequent bladder surgery is directly related to the presence or absence of ipsilateral lower pole or contralateral reflux. Husmann et al. reported a definitive cure in only 16% of patients in this setting if endoscopic decompression or an upper-tract approach was used alone. In fact, the need for additional surgery was related to the number of renal moieties with reflux at presentation, reporting a 96% reoperative rate with unilateral high-grade reflux or reflux seen in more than one renal moiety [16].

In conclusion, ectopic ureteroceles that reflux or are associated with reflux into other moieties are likely best served with ureterocele excision or marsupialization, bladder floor reconstruction, and ureteral reimplant. Another option would be a ureteroureterostomy with a lower-pole extravesical reimplant. In those patients who present with sepsis or bladder outlet obstruction, endoscopic decompression is highly successful but will likely commit the patient to further surgery. Upper-pole heminephrectomy is best applied to those patients with nonfunctioning upper poles and no associated reflux. In this setting, this approach is highly successful and has the advantage of avoiding bladder surgery, limiting risks to the lower pole, and eliminating the potential unknown risks of preserving a dysplastic upper pole [15]. Arguably, upper-pole heminephrectomy can be performed open or laparoscopically.

## 3. CONCLUSIONS

Reflux found in association with a duplex system may take longer to resolve than single-system reflux. Parents should be counseled accordingly. Surgery to correct VUR in duplex systems is highly successful. Ectopic ureteroceles can present an interesting and difficult surgical challenge and can be ultimately managed with multiple surgical approaches following initial conservative therapy. Endoscopic decompression seems best reserved for the septic patient or one who presents with bladder outlet obstruction. It provides excellent relief of obstruction and can preserve upper-pole renal function. Ultimately, these patients are currently managed by either an upper- or lower-tract approach. The most important factor in deciding which approach to take is the presence or absence of VUR.
